# Pulsed Radiofrequency Ablation for Refractory Cancer-Related Leg Pain: A Case Report

**DOI:** 10.7759/cureus.58779

**Published:** 2024-04-22

**Authors:** Praveen Reddy Elmati, Tyler J Wilkinson, Alexander Bautista

**Affiliations:** 1 Anesthesiology, Beebe Hospital, Georgetown, USA; 2 Anesthesiology, University of Louisville Hospital, Louisville, USA

**Keywords:** opioid-sparing, palliative care, femoral nerve involvement, peritoneal carcinomatosis, gastric adenocarcinoma, neuropathic leg pain, dorsal root ganglia, pulsed radiofrequency ablation

## Abstract

Metastatic cancers often lead to distant metastasis, accompanied by debilitating symptoms such as chronic pain, which can be refractory to conventional analgesic modalities. Pulsed radiofrequency ablation (Pulsed RFA) has emerged as a promising intervention for neuropathic pain syndromes, offering long-lasting relief with minimal tissue damage. We present a case of a 36-year-old male with metastatic gastric adenocarcinoma and refractory leg pain due to femoral nerve involvement. Despite aggressive multimodal analgesia, the patient experienced persistent pain, necessitating alternative interventions. Pulsed RFA targeting the right L2-L4 dorsal root ganglia (DRG) provided significant and sustained pain relief, allowing improved functional status and reduced opioid requirements. This case underscores the potential of pulsed RFA as an effective intervention for refractory cancer-related pain, enhancing patients' comfort and quality of life. Further research is warranted to establish its long-term efficacy and safety.

## Introduction

Metastatic gastric adenocarcinoma frequently leads to peritoneal carcinomatosis, accompanied by debilitating symptoms including chronic pain. Conventional pain management approaches, which include opioid analgesics and nerve blocks, may provide temporary relief but often fail to offer sustained benefits [1]. Severe pain often requires multimodal management to provide relief. This necessitates the exploration of alternative interventions that target the underlying mechanisms of pain transmission.

Pulsed radiofrequency ablation (RFA) delivers radiofrequency current in short and high voltage repeated electrical bursts. The resting phase allows for heat elimination thereby keeping the target tissue temperature below 42° C. It reduces the functioning of the neuron synapses in c fibers and inhibits pain signal transmission from the peripheral nerve to the central nervous system. It is also postulated to deactivate microglia at the spinal dorsal horn, decrease immune cells, and proinflammatory cytokines, and increase endogenous opioid precursor messenger ribonucleic acid. It is reported to decrease levels of interleukin 1. Metalloproteinase -3 and tumor necrosis factor-alpha in the tissues after treatment [2]. It can also enhance the noradrenergic and serotonergic pain inhibitory pathway and damage nociceptive C and A delta fibers affecting their functioning [3, 4].

Pulsed RFA has emerged as a promising technique for managing neuropathic pain syndromes, offering the potential for long-lasting relief without the risks associated with conventional ablative procedures [5, 6]. Pulsed RFA modulates pain signaling pathways while minimizing tissue damage [7, 8].

Informed consent was obtained before publication of this case report. We describe the successful utilization of pulsed RFA targeting the right L2-L4 dorsal root ganglia (DRG) to alleviate refractory right leg pain in a 36-year-old male with peritoneal carcinomatosis, femoral nerve involvement, and metastatic gastric adenocarcinoma. 

## Case presentation

**Figure 1:**
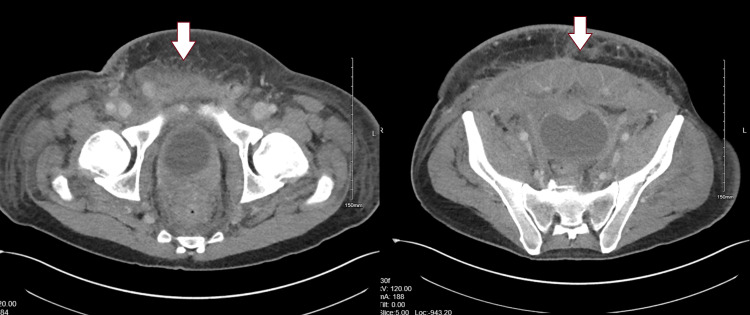
CT of the pelvis in the axial plane showing metastatic gastric adenocarcinoma along the anterior pelvic wall.

A 36-year-old male presented with a 6-month history of progressive abdominal pain, intractable leg pain, nausea, and vomiting. Imaging studies revealed stage IV gastric adenocarcinoma with peritoneal carcinomatosis and metastases to the abdomen, skin, and right thigh. The patient reported chronic pain in the abdomen and right lower extremity (LE), attributed to tumor involvement of the abdominal wall and femoral nerve. The patient's pain was 7-8 out of 10 on a numerical pain rating scale. The patient underwent palliative radiation therapy for the tumor around the umbilical area. Repeat imaging studies showed the tumor to be stable without progression. Since the cancer was metastatic and non-responsive to treatment, the patient opted for palliative care. Figure 1 shows the spread of gastric adenocarcinoma along the anterior abdominal wall. Chronic pain management was consulted for severe right leg pain affecting ambulation.

Despite aggressive multimodal analgesia, including patient-controlled analgesia (PCA), IV hydromorphone, and transdermal fentanyl patches (100 mcg), the patient experienced persistent severe leg pain and abdominal wall pain refractory to conventional therapies. The patient was unable to tolerate oral pain medications due to underlying gastric cancer and his risk of recurrent gastrointestinal bleeding. A femoral nerve block provided partial relief for 2 weeks necessitating alternative interventions to improve pain control and provide comfort. The patient refused the placement of an intrathecal pain pump for pain control.

**Figure 2:**
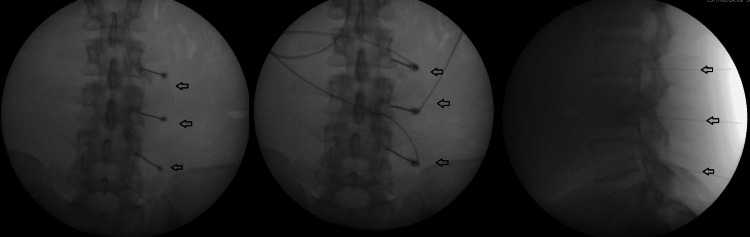
X-ray-guided imaging of right L2-L4 dorsal root ganglion Left and Middle images are anterior-posterior views with a radiofrequency ablation probe; Right image is a the lateral view.

Given the limited efficacy of conventional analgesic modalities, the patient underwent evaluation for pulsed RFA targeting the right L2-L4 dorsal root ganglion (DRG). After obtaining informed consent, the procedure was performed under fluoroscopic guidance. Under strict aseptic conditions, percutaneous radiofrequency needles were inserted adjacent to the targeted DRG using fluoroscopic visualization. Sensory and motor stimulation confirmed the appropriate positioning of the needles, followed by the delivery of pulsed radiofrequency energy at 42°C for 120 seconds. The procedure was well-tolerated without any immediate complications. Figure 2 shows pulsed radiofrequency ablation of the right L2-L4 dorsal root ganglion 

Post-procedure, the patient reported a significant reduction in leg pain intensity, with >80% improvement with pain relief. The patient stated pain of 1-2 out of 10 on a numerical pain rating scale. Remarkably, he no longer required hydromorphone PCA after the pulsed RFA. Pain relief from pulsed RFA enhanced the patient's functional status and overall well-being by allowing engagement in daily activities, permitting ambulation, and reducing opioid-related side effects. The patient was placed on comfort care and passed away a week later. The successful outcome of pulsed RFA highlights its potential as an effective intervention for refractory cancer-related pain, offering patients improved comfort and quality of life.

## Discussion

Pulsed RFA of DRG represents a novel approach to the management of refractory neuropathic pain syndromes, including those secondary to cancer metastasis [9,10]. In this case, pulsed RFA targeting the right L2-L4 DRG provided significant and sustained relief of intractable leg pain in a patient with metastatic gastric adenocarcinoma with anterior abdominal wall and femoral nerve involvement. By selectively modulating pain transmission without causing irreversible tissue damage, pulsed RFA offers a safe and effective alternative for patients with limited treatment options. The patient was able to ambulate post-procedure with decreased pain and discomfort. The patient's refusal of a pain pump highlights the importance of considering patient preferences and individualized treatment approaches in the management of cancer-related pain. Further research is warranted to elucidate the long-term efficacy, safety profile, and optimal patient selection criteria for pulsed RFA in palliative care. Additionally, multidisciplinary collaboration among pain specialists, oncologists, and palliative care teams is essential to individualize treatment approaches and optimize symptom management in patients with advanced cancer.

In a retrospective observational study by Cassini et al on Pulsed RFA in a case series of 31 patients in Europe post-procedural follow-up after a median of 119 days most patients reported significant improvement in pain control. At the last follow-up around 75% of patients continued to have significant pain relief thus proving to be a promising procedure for pain management [11]. Facchini et al in their systematic review of Pulsed RFA describe it as a promising procedure for cervical radicular pain and sacroiliac joint pain based on current evidence [12].

Facchini et al also describe the complications of the procedure, including local swelling post-procedure, rare occasions of neural trauma, injection into blood vessels, hematoma, injury to local nerves including sciatic nerve, and abscess formation in the intra-articular region. Minor complications such as lightheadedness, flushing, nausea, syncope, and low blood pressure post-procedure were reported by the authors [12].

## Conclusions

Pulsed RFA of the dorsal root ganglion represents a valuable adjunctive therapy in the multimodal management of cancer pain. With its favorable safety profile and potential for significant pain relief, pulsed RFA holds promise in improving the quality of life and symptom control in patients with advanced malignancies. 
